# Photocatalytic activity of layered MoS_2_ in the reductive degradation of bromophenol blue†

**DOI:** 10.1039/d2ra03362c

**Published:** 2022-08-11

**Authors:** Joanna Kisała, Ana M. Ferraria, Nataliya Mitina, Bogumił Cieniek, Piotr Krzemiński, Dariusz Pogocki, Roman Nebesnyi, Oleksandr Zaichenko, Yaroslav Bobitski

**Affiliations:** Department of Biology, Institute of Biology and Biotechnology, University of Rzeszow Pigonia 1 35-310 Rzeszow Poland jkisala@ur.edu.pl; BSIRG-iBB-Institute for Bioengineering and Biosciences, Universidade de Lisboa 1049-001 Lisbon Portugal; Associate Laboratory i4HB—Institute for Health and Bioeconomy at Instituto Superior Técnico, Universidade de Lisboa 1049-001 Lisboa Portugal; Department of Organic Chemistry, Institute of Chemistry and Chemical Technologies, Lviv Polytechnic National University 79013 Lviv Ukraine; Institute of Materials Science, College of Natural Sciences, University of Rzeszow Pigonia 1 35-959 Rzeszow Poland; Centre for Microelectronics and Nanotechnology, Institute of Physics, University of Rzeszow Pigonia 1 35-959 Rzeszow Poland; Institute of Nuclear Chemistry and Technology Dorodna 16 03-195 Warsaw Poland; Technology of Organic Products Department, Lviv Polytechnic National University 12S. Bandera St. Lviv 79013 Ukraine; Department of Photonics, Lviv Polytechnic National University 1 Sviatoho Yura Sq. 79013 Lviv Ukraine

## Abstract

Molybdenum disulphide (MoS_2_) is a layered material with interesting photocatalytic properties. In this study, a layered MoS_2_ was produced using a hydrothermal method. The obtained material was characterised by XRD (X-ray diffraction), XPS (X-ray photoelectron spectroscopy), SEM (scanning electron microscopy), UV-Vis spectroscopy, DLS (dynamic light scattering), and zeta potential analysis. For the evaluation of the photocatalytic properties of layered MoS_2_, a solution of bromophenol blue (BPB) and the catalyst was illuminated for 120 minutes. According to the experimental results, MoS_2_ exhibited excellent catalytic activity in BPB degradation. The MoS_2_ preparation method enabled improved light harvesting, avoided fast charge recombination (related to bulk MoS_2_), and created a large number of suitable electron transfer sites for photocatalytic reactions. Simulation of BPB decay and bromide production was carried out for a further understanding of MoS_2_ photocatalytic action. The simulation results proved the reduction mechanism of BPB photodegradation.

## Abstract 2

Molybdenum disulphide (MoS_2_) is a layered material with interesting photocatalytic properties.

## Graphical abstract

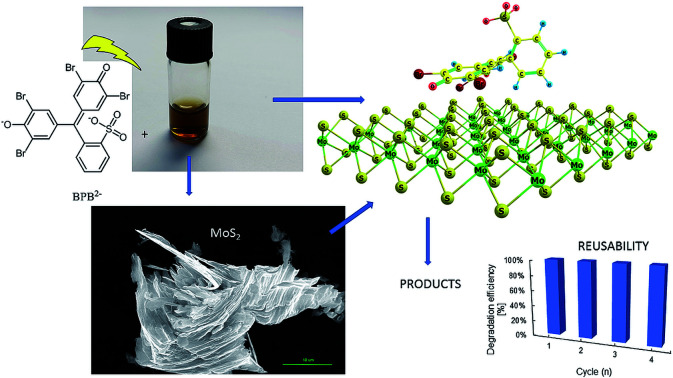

## 1. Introduction

In 2019, the WHO (World Health Organisation) declared that over 2.2 billion humans do not have access to clean water. Continuous shrinking reserves of global drinking water are caused in part by water pollution. Water contamination, causing serious environmental and health problems, has gained a lot of attention worldwide. The low-cost wastewater decontamination would be a partial remedy for the emerging crisis. A meeting of the demands of environmental remediation is a great challenge for society, especially for scientists, researchers, and process engineers.

The wastewater from the textile industry, even with very low dye concentrations, can intensely colour the wastewater. Apart from negative visual effects, such aromatic compounds are highly non-biodegradable and mostly carcinogenic and mutagenic.^1^ Usage of renewable energy sources, *i.e*., solar energy, in the photocatalytic process is one of the efficient methods allowing the degradation of environmental pollutants caused by the industry.

Bromophenol blue (BPB) is an acid dye applied as a colour marker to monitor the process of agarose gel electrophoresis, polyacrylamide gel electrophoresis, for colouring proteins in paper electrophoresis, and as a pH indicator. This dye is widely used as an industrial dye for foods, drugs, cosmetics, textiles, printing inks, and as a laboratory indicator.^2^ Additionally, BPB is a convenient model compound for the investigation of the photodegradation of structurally similar polyhalogenated bisphenols extensively used as flame retardants.^3–5^

Molybdenum disulphide (MoS_2_), used here as a photocatalyst, is a semiconducting material with a layered structure, belonging to the family of transition metal dichalcogenides.^6–8^ Solid molybdenum disulphide (MoS_2_, molybdenite) is a chemically stable layered material, made up of two layers of sulphur atoms and a layer of molybdenum atoms, forming a sandwich-like structure.^9^ Due to its peculiarity, it is widely used in the photoelectric conversion and as a photocatalyst.^10^ Actually, it is a small-particle semiconductor with a small band gap, allowing valence-band electrons to be excited to the conduction band by almost the entire range of the visible light spectrum. Additionally, the single-layer MoS_2_ possesses excellent charge carrier mobility, as good as the carbon nanotube. The sheet structure has a superior surface-to-volume ratio that could be an advantage for catalysing materials.

Advanced oxidation processes (AOPs) have received a lot of attention in the literature in recent years. However, not all compounds can be effectively oxidised because, in fact, they already are in an oxidised form. This applies, for example, to organic compounds containing halogens.^11,12^ Therefore, the need for an alternative to *in situ* oxidation processes has been recognised. Advanced reduction processes (ARPs) rely on the production of strongly reducing hydrated electrons (e_aq_^−^), which exhibit fast reaction rate constants with inorganic and organic compounds.

There is very little research available on the application of ARPs in the degradation of organic water pollutants. Current research is one of the few on this topic. In this paper, the authors present the synthesis of MoS_2_ multilayer nanostructures by the hydrothermal method. Their morphology and structure were characterised by scanning electron microscopy (SEM), X-ray diffraction spectroscopy (XRD), atomic force microscopy (AFM), and Raman spectroscopy. The photocatalytic properties of MoS_2_ in the degradation of bromophenol blue (BPB) as a model pollutant were assessed.

## 2. Experimental section

### 2.1 Materials

Li_2_MoO_4_ was used as the molybdenum source and Na_2_S was used as the sulphur source to prepare MoS_2_. Lithium molybdate (Li_2_MoO_4_), sodium sulphide (Na_2_S), and hydrazine dihydrochloride (N_2_H_4_ 2HCl) were purchased from Sigma-Aldrich (St Louis, MO, USA).

The hydrogen ion concentration of the investigated systems was adjusted by NaOH and HCl. Bromophenol blue (St Louis, MO, USA) was used as a representative dye to evaluate the photocatalytic properties of layered MoS_2_. *Tert*-butanol was used in the experiment as the hydroxyl radical scavenger. All reagents were of analytical grade and were used as received.

### 2.2 Preparation of MoS_2_

The synthesis of MoS_2_ nanoparticles was carried out: at the first stage 5.4 g of Li_2_MoO_4_ was dissolved in 48.4 mL of 20% Na_2_S aqueous solution. After 30 minutes of constant stirring, Li_2_MoS_4_ precipitated under the reaction:

1 Li_2_MoO_4_ + 4Na_2_S + 4H_2_O → Li_2_MoS_4_ + 8NaOH

Solid Li_2_MoS_4_ was mixed with a solution of hydrazine chloride (0.62 M L^−1^) and the pH value of the reaction medium was adjusted to 7.8 by the addition of 0.1 M L^−1^ HCl solution. Afterwards, the suspension was inserted into the Teflon coated autoclave and heated for 24 h at 473 K. The resulting precipitate of black colour was washed out with distilled water three times and finally with ethyl alcohol. The solid was separated by centrifugation and dried at 313 K.

### 2.3 Photocatalyst characterisation

The morphology and particle size of prepared nanoparticles were evaluated by a field emission scanning electron microscope (FESEM) Helios NanoLab 650 (FEI, Hillsboro, Oregon, USA) operating at 5 kV and 18 kV using ETD detector with Secondary Electron (SE) imaging mode. The MoS_2_ nanoparticles were mounted on a specimen table using conductive carbon tape. Images were taken at different magnifications and measurements were conducted on visibly separated nanoparticles. The specimen was uncoated.

Atomic force microscopy (AFM) measurements were performed using a Solver Nano II Microscope (NT-MDT Spectrum Instruments LLC, Tempe, Arizona, USA). The solution of the MoS_2_ was deposited on the substrate following the drop casting method. The samples were evaporated in desiccator during 24 hours. Evaporated sample was analysed using tapping mode.

XRD patterns were recorded with an X-ray diffractometer (D8 Advance, Bruker, Germany) and 0.15406 nm Cu K_α_ radiation. The average crystallite sizes (*D*) of MoS_2_ samples were calculated from the powder XRD line widths by applying the Debye–Scherrer eqn (2)^13,14^2
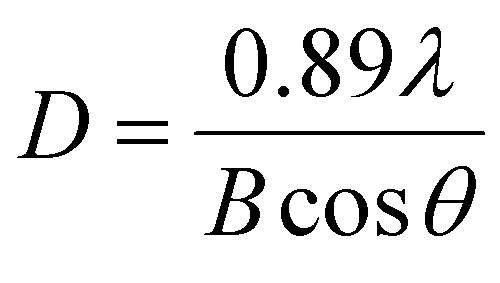
where *λ* is the wavelength of the X-ray in nanometres, *B* is the peak width at half-height (FWHM), and *θ* is the angle between the incident and diffracted beams in angular degrees.

The Raman spectra were obtained using an inVia Micro Raman Renishaw spectrometer combined with a Leica DM 2500 M microscope (Renishaw, Wotton-under-Edge, UK) equipped with a 633 nm laser as an excitation source. The measurements were taken with an exposure time of 10 s with triple scan accumulation and for the laser output power of 1.5 mW. The data were collected in the spectral range of 100–1500 cm^−1^ with spectral resolution over 2 cm^−1^. The measurement was carried out with a 50× lens magnification and choosing five random positions. Baseline correction was performed during data processing.

The powdered MoS_2_ sample was analysed by X-ray photoelectron spectroscopy (XPS) with a dual anode non-monochromatic XSAM800 spectrometer from KRATOS (Kratos Analytical Ltd, Manchester, UK). Al K_α_ X-rays (*hν* = 1486.6 eV) were used. The acquisition conditions were as published elsewhere.^15^ The MoS_2_ powder was fixed with a silicone-based double face tape, completely covering the XPS holder and analysed at TOA = 45°. No flood gun was used for charge neutralisation. The charge shift was corrected using as a reference the binding energy (BE) of sp^2^ C–C and/or C–H, centred at 284.7 eV. Source satellites were subtracted. Pseudo-Voight profiles (Gaussian–Lorentzian (GL) products) were used for peak fitting with the XPSPeak4.1 software (freeware). Shirley baselines were used. The sensitivity factors used for the quantification analysis were those of the library of Vision 2 for Windows, Version 2.2.9 from KRATOS.

The optical measurements of the catalysts were carried out using an Agilent Technologies Cary Series UV-Vis-NIR spectrophotometer (Agilent, Santa Clara, CA, USA) in the wavelength range of 180 to 1200 nm. The UV-Vis spectra of organic compounds solutions were measured on a VWR UV-VIS 3100 PC spectrophotometer (VWR International Ltd, Gdansk, Poland).

The hydrodynamic diameter of the semiconductor particles and the electrokinetic potential (zeta potential, *ζ* potential) were measured as a colloidal dispersion with a concentration of 1.25 × 10^−1^ g L^−1^. The zeta potentials were determined by electrophoretic mobility measurement in a particle suspension. The hydrodynamic diameter of the catalysts was measured by Dynamic Light Scattering (DLS). These measurements were performed with the NanoPlus 3 HD analyser (Particulate Systems, Micromeritics, Norcross, GA 30093, USA).

### 2.4 Photocatalytic experiment conditions

The photocatalytic activity of the MoS_2_ catalyst was evaluated by monitoring the degradation of bromophenol blue (BPB). 250 mL of 1.0 × 10^−4^ M L^−1^ BPB solution, 0.05 g of catalyst powder, and 0.3 M L^−1^*t*-BuOH were placed in the photoreactor. The resulting suspension, pH 5.2, was then stirred for 30 minutes in the dark under argon. The photocatalytic degradation was performed using a Heraeus LRS2 photoreactor in a continuous argon flow. The illumination was effected with the excimer lamp TQ150 (150 Watt, with forced water cooling down to 25 °C, of 47 W light energy flux of power density 4.696 mW cm^−2^ measured by digital lux meter Peak Tech 5025) placed in a vertically oriented dip tube, immersed in the continuously agitated reaction mixture. The photocatalytic reaction was performed up to 120 minutes illumination time. During the reaction, 2 mL samples were collected from the reactor at regular time intervals, when the concentration of compounds was monitored using a UV-Vis spectrophotometer with prior removal of the solid catalyst.

The total solubilised bromide (Br^−^) was determined potentiometrically using a multimeter (CPC 411, Elmetron, Poland) equipped with a bromide ion-selective electrode (EBr-01, Hydromet, Poland) with the silver chloride electrode (RL-100, Hydromet, Poland) as a reference electrode.

We performed reusability tests of the catalyst for a four cycles of photocatalysis. The degradation BPB efficiency calculation equation is shown in eqn (3).3
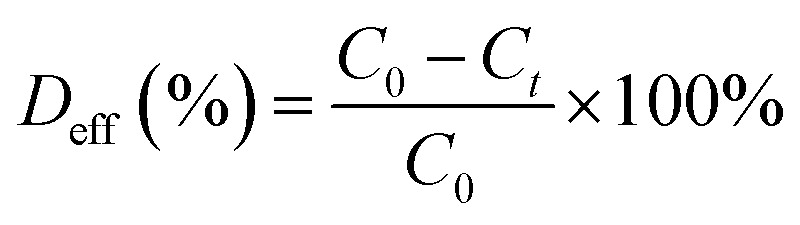


*C*_0_ is the initial concentration of BPB, and *C*_*t*_ is the concentration at time *t* (min).

### 2.5 Kinetic simulations

Competitive kinetics simulations were performed with the Kinetiscope™ stochastic kinetic simulation software^16–18^ freely available on the authors' web page: https://www.hinsberg.net/kinetiscope/. The rate constants of the radical reactions describing the investigated process, required for the competitive kinetics simulation, were taken from the compilation of the rate constants^19^ and shown in Table S2.†

## 3. Results and discussion

### 3.1 Photocatalyst characterisation

MoS_2_ powders of different morphologies have been obtained using a wide variety of methods such as thermal decomposition of ammonium tetrathiomolybdate or amorphous MoS_3_,^20^ high-temperature reaction of the stoichiometric mixture of molybdenum and sulfur powders under vacuum,^21^ gas-phase reactions of H_2_S and molybdenum oxides under reducing atmosphere.^22,23^ There are also reports on high energy synthetic procedures using laser,^24^ electron beam,^25^ and gamma radiation^26^ leading to molybdenum disulphide as nanotubes, fullerene-like, and other curved nanostructures. The solution-based synthesis procedure developed in this paper is simple and convenient could be an alternative to the methods mentioned above. Furthermore, lithium ions present in the reaction mixture can intercalate and interlayer spacing of MoS_2_.^27^ For this reason, the obtained MoS_2_ material has semiconductor properties.

**Fig. 1:**
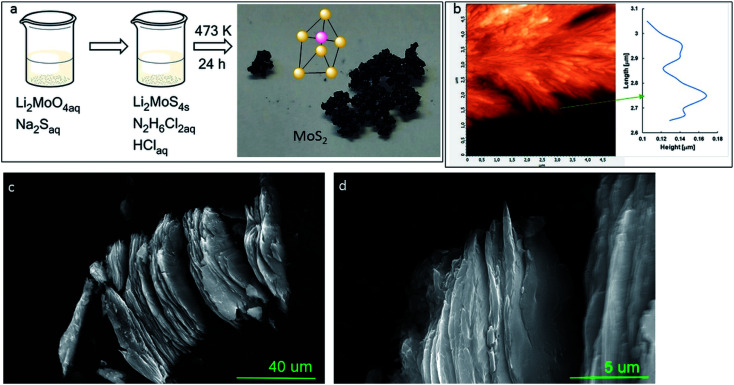
Schematic description of MoS_2_ synthesis method (a), AFM image and height profile (b), SEM images of two different points and different magnifications of MoS_2_ (c and d).

SEM micrograph (Fig. 1c and d) reveals that the material has a layered structure. The topography of MoS_2_ was evaluated by AFM measurements. The AFM image shown in Fig. 1b reveals a layered structure of the material with a thickness range of 100–160 nm.

**Fig. 2:**
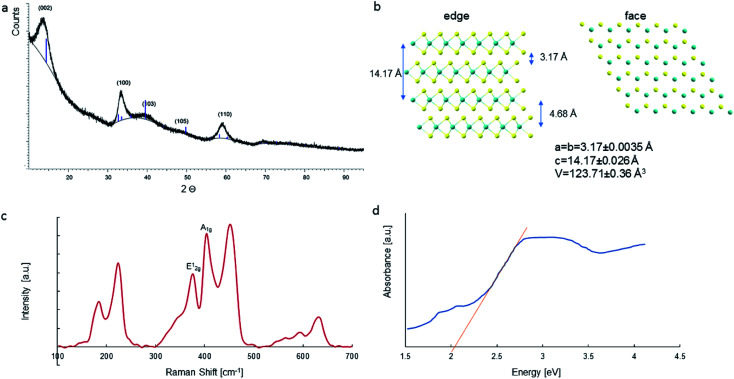
XRD pattern of the MoS_2_ sample and (a); Top and side view of MoS_2_ with the crystal parameters (b); Raman spectra of MoS_2_ (c); UV-Visible absorption spectra (d).

The obtained MoS_2_ was analysed using XRD measurements (Fig. 2a). The resulting peaks are characteristic for 2H–MoS_2_ (JCPDS card no 01-087-2416, *P*6_3_/*mmc*)^28^ with lattice parameters *a* = 3.17 ± 0.0035 Å and *c* = 14.17 ± 0.026 Å. The unit cell is presented in Fig. 2b. Each layer of MoS_2_ has a plane of hexagonally arranged molybdenum atoms sandwiched between two planes of hexagonally arranged sulfur atoms, with the covalently bonded S–Mo–S atoms in a trigonal prismatic arrangement form a hexagonal crystal structure. The XRD results represented that the obtained materials have a pure phase. Five diffraction peaks were observed, which can be assigned to the (002), (100), (103), (105), and (110) planes of MoS_2_, the 2θ values are 13.95; 33.3; 39.8; 48.2; and 59.2, respectively. The peak broadening indicates a sheet-like crystallite morphology with an average size of 50 nm. The XRD spectrum of the MoS_2_ is comparable to that reported by Quilty *et al.*,^29^ and Ortis-Quiles and Cabrera.^30^ It confirms that the received material is layered. Layered MoS_2_ generally displays n-type behaviour. The *d*_002_ line observed at 2*θ* = 13.95 Å showed a shift towards a lower angle regarding bulk 2H–MoS_2_. The observed shift may be caused by the lattice expansion compared to that report for 2H–MoS_2_ (*a* = 3.16 Å, *c* = 12.29 Å). This lattice expansion may be ascribed to the folding and randomness in the stacking of the MoS_2_ layers.^31^

The optical properties of MoS_2_ were analysed using the UV-Vis spectra (Fig. 2d). The dye degradation efficiency depends on the energy of the photocatalyst energy gap. When using visible light, a photocatalyst with a lower energy gap energy is preferable. The material exhibited a good optical response to ultraviolet and visible light, which was ascribed to its narrow band gap. The band gap width of 2.03 eV was determined from the measurements of the MoS_2_ absorption spectrum. In addition, absorption peaks at 1.88 eV (659 nm) and 2.08 eV (596 nm) were observed in the spectrum due to the division of the orbit transitions by the spin–orbit coupling.^32,33^ The absorption of visible light consequently causes the formation of holes in the valence band by the promotion of electrons to the conduction band.

The recorded Raman spectra contain several bands distinct to the MoS_2_ material (Fig. 2c). In the spectra, was observed peaks at 375, 404, and 451 cm^−1^, which correlate with the phonon mode of 2H–MoS_2_. The peak of 2H MoS_2_ at 378 cm^−1^ corresponds to E_2g_^1^, due to the opposite vibration of two S atoms regarding the Mo atom, the peak at 404 cm^−1^ is attributed to A_g_^1^, associated with the vibration of only S atoms in opposite directions and at 451 cm^−1^ is ascribed to longitudinal acoustic phonon modes.^34^ These results demonstrate the 2H–MoS_2_ structure of the tested material.

Raman spectroscopy may be used for estimating the average number of MoS_2_ layers. It is known that the distance between the in-plane S atom (E_2g_^1^) and out-of-plane S atom (A_1g_) vibrational modes corresponds to the crystal thickness. The distance between the E_2g_^1^ = 378 cm^−1^ and A_1g_ = 404 cm^−1^ vibrational modes was measured, and it was 26 cm^−1^, representing ≤5 layers.^35^

Absorbance peaks of UV-Vis spectrum at 659 and 596 were utilised as references for determining the particle size and number of MoS_2_ layers using method previously successfully applied by:^36,37^4
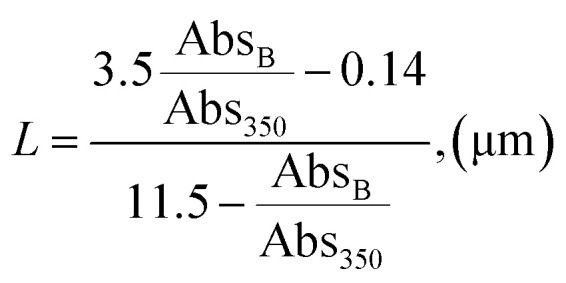

5 *N* = 2.3 × 10^36^e^−54888/*λ*_A_^

where: Abs_B_ is the absorbance of peak at 596 nm and Abs_350_ is the absorbance of peak at 350 nm in the UV-Vis spectrum.

The calculated particle lateral size (*L*) was 137 nm, while the number of layers was estimated as <5, correlating with Raman characterisation.

**Fig. 3:**
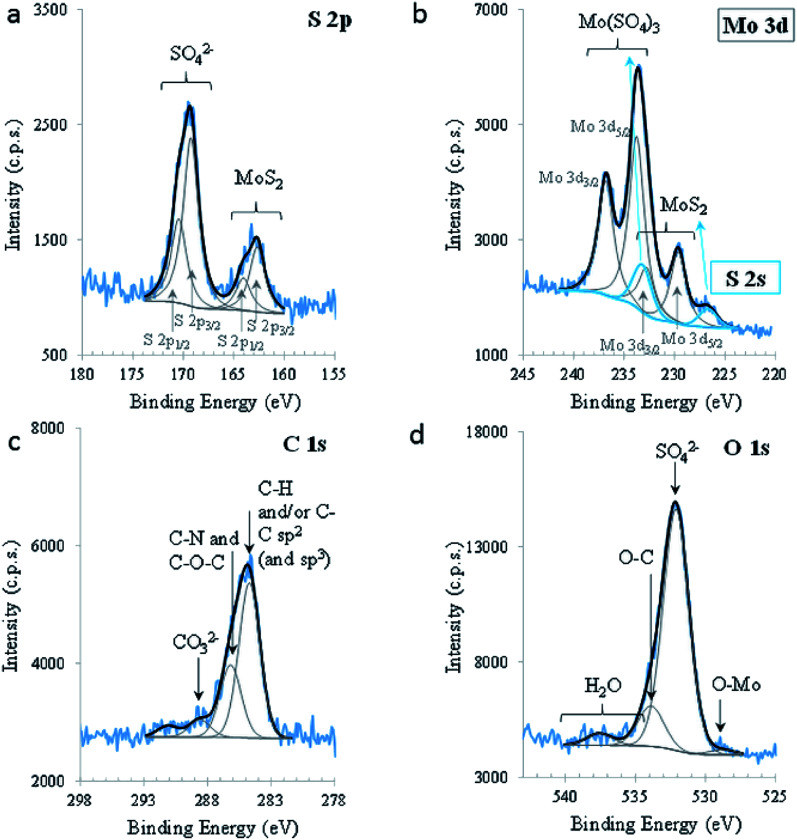
XPS regions S 2p (a), Mo 3d (and S 2s) (b), C 1s (c), and O 1 s (d).

To investigate the chemical composition and the nature of the chemical bonds between the elements present in MoS_2_, XPS was carried out. Fig. 3 shows the relevant XPS regions. S 2p shows clearly the presence of two doublets, with a spin–orbit split (SOS) of 1.3 ± 0.1 eV, with the main components (*i.e.*, S 2p_3/2_ components) centred at 162.6 ± 0.1 eV and 169.2 ± 0.1 eV, assigned to S in MoS_2_ and in SO_4_^2−^ groups, respectively. The oxidation of S_2_^−^ in the alkaline medium is predicted by the reaction:

6 SO^2−^_4(aq)_ + 8e^−^ + 4H_2_O_(l)_ ↔ S^2−^_(aq)_ + 8OH^−^_(aq)_

Mo 3d is also composed of doublets which, in this case, are overlapping the S 2s region (blue peaks). The peak centred at lower BE (226.7 ± 0.1 eV) is attributed to sulphur (S 2s) in 2D-MoS_2_. Another peak, centred at 233.2 eV and assigned to sulphur in sulphate groups, was fitted in this region, constraining its area to the same proportion of sulphate/sulphide found in S 2p. Its position was not restricted. Mo 3d is composed of two doublet peaks, with SOS between Mo 3d_5/2_ and Mo 3d_3/2_ equal to 3.1 (0) eV and a ratio (3d_5/2_)/(3d_3/2_) equal to 1.5; these parameters were imposed for both doublets, leaving Mo 3d_5/2_ positions, areas, FWHM and GL free to fit. Mo 3d_5/2_ centred at 229.6 ± 0.1 eV and 233.7 ± 0.1 eV are assigned to Mo^4+^ in 2D-MoS_2_ and to Mo^6+^, respectively. Mo^6+^ is mainly under the form of Mo(SO_4_)_3_ containing also some MoO_3_, as attested by the O^2−^ peak, centred at 528.8 eV in O 1s, and Mo(CO_3_)_3_, confirmed by the presence of a peak at 288.6 eV in C 1s. The existence of these species is also confirmed by the quantitative analysis (Table S1†). Oxidation to Mo^6+^ in an alkaline medium is predicted by the reaction:

7 MoO^2−^_4(aq)_ + 2e^−^ + 2H_2_O_(l)_ ↔ MoO_2(s)_ + 4OH^−^_(aq)_

The valence band (VB) extracted from the XPS spectrum was also analysed. The energy levels are referred to the Fermi level (*E*_F_) stated in Fig. S1.† The top of the VB is 1.6 eV, apart from the *E*_F_. The fine spectral structure perceived between the top of VB and *E*_F_, may be assigned to the density of states. The XPS results confirmed the behaviour of MoS_2_ semiconductors as n-type.

**Fig. 4:**
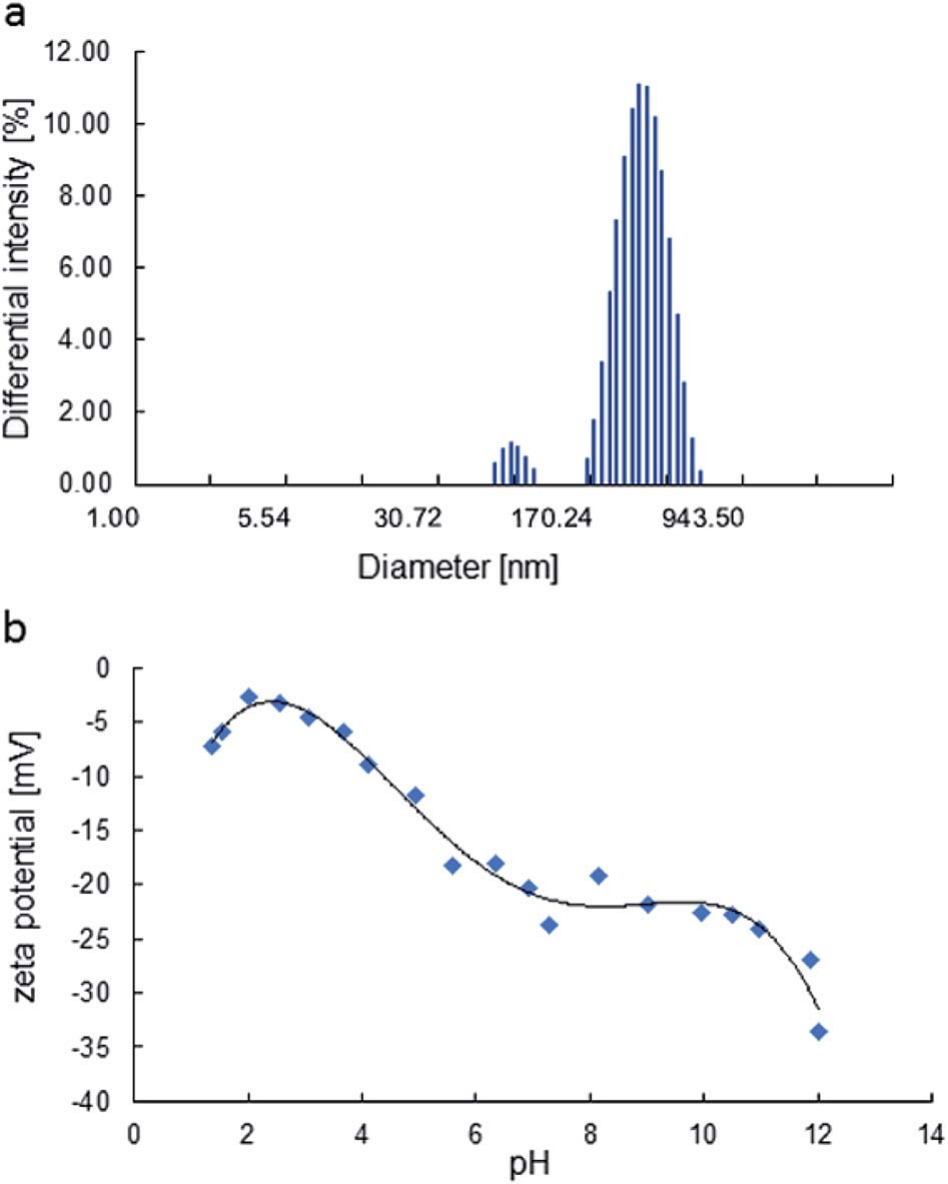
The particle size distribution of the H_2_O dispersion, obtained by DLS (a), zeta potential of MoS_2_ as a function of pH (b).

The particle size of MoS_2_ in H_2_O was also evaluated by DLS (considering their hydrodynamic radius), where the platelet sizes were ranging from 72 to 564 nm (Fig. 4 a). The zeta potential dictates the sign and amount of surface charge in relation to the surrounding conditions. The zeta potentials were negative for all ranges of pH (Fig. 4 b) and decrease with pH. The face/edge ratio (hydrophobic – sulphur surface/metallic – hydrophilic edge) strongly influences the magnitude of the zeta potential being inversely proportional.^38^

The zeta potential of molybdenite particles is due to the generation of electric charges at the edges of the particles, since the faces present only van der Waals forces and no electric charge.^39^ The processes on the molybdenite edges can be described:8
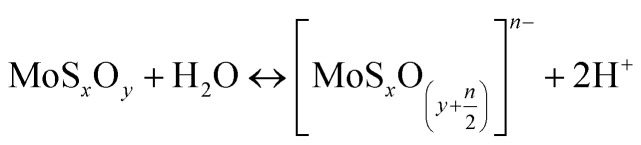
9
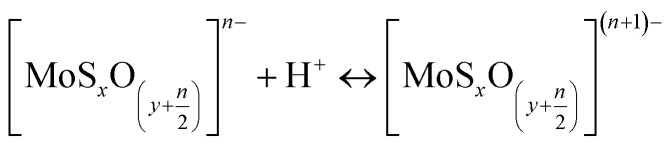


These reactions explain the pH decrease of molybdenite powders dispersed in water. The next equation describes the process under alkaline conditions.

10 HMoO^−^_4_ ↔ H^+^ + MoO^2−^_4_, p*K*_a_ = 5.95

It explains the high negative values of zeta potential in high pH conditions.

Wan *et al.*^40^ reported that the zeta potential of molybdenite was mainly determined by edges and not faces, especially in the case of fine particles. Compared to the hydrophobic surfaces, the edges of the molybdenite oxidised more easily in the solution.

### 3.2 Evaluation of photocatalytic properties

**Fig. 5:**
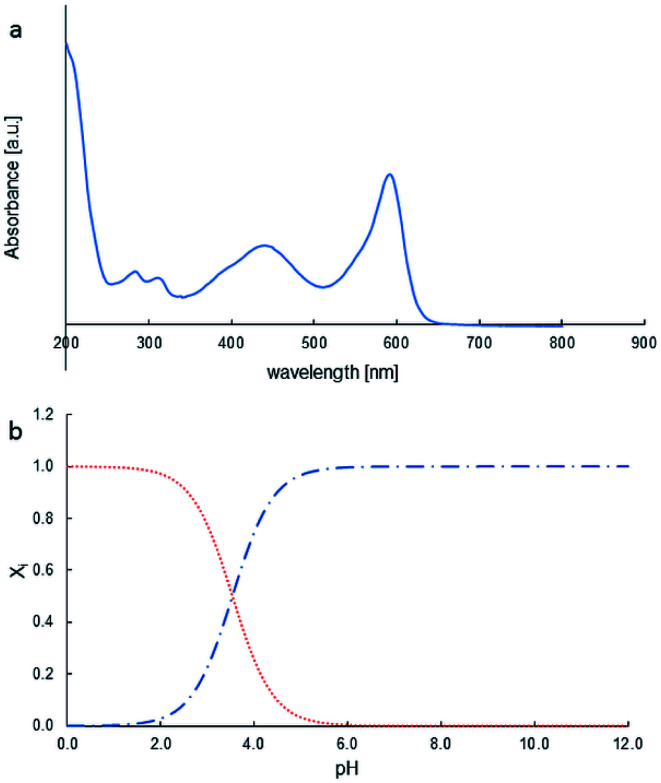
BPB UV-Vis spectrum (a); distribution of species as pH function, BPB^−^ (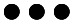 red), BPB^2−^ (
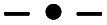
 blue) (b).

The photocatalytic properties of MoS_2_ materials depend on a variety of parameters, such as particle size, number of layers, and the face/edge ratio. The photocatalytic properties of MoS_2_ were tested in bromophenol blue (BPB) degradation. Fig. 5a presents the BPB absorption spectrum. Diagnostic was the peak at 590 nm for dye decay monitoring.

**Fig. 6:**
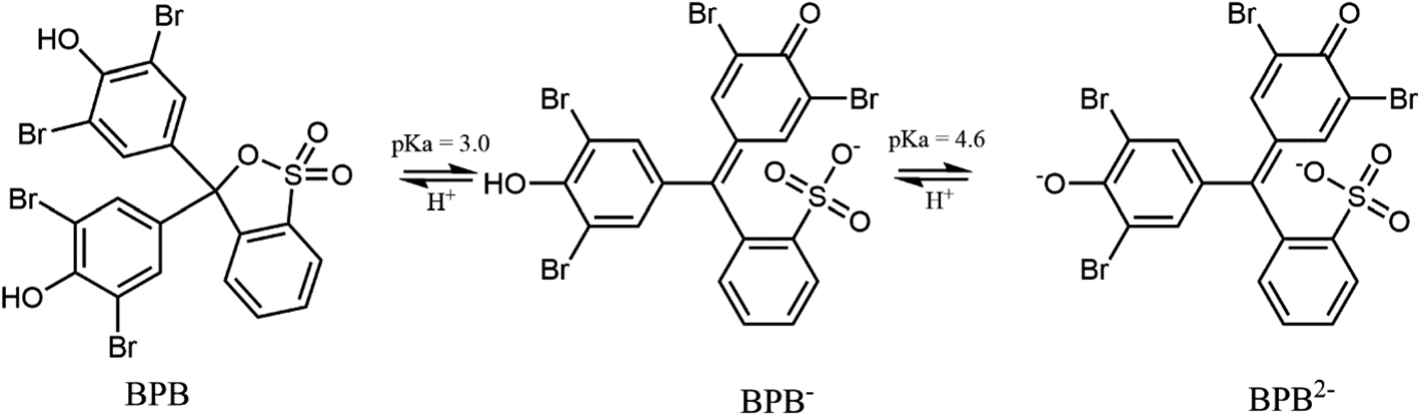
Dissociation equilibrium of the pH indicator BPB.

The photocatalytic activity tests of the synthesised MoS_2_ were evaluated by carrying out the degradation of bromophenol blue in weak acidic aqueous solution (pH 5.2). Under such conditions, BPB is present in solution only in the fully dissociated form; the respective first and second acid dissociation constants, pKa, are 3.00 and 4.6 (Fig. 5b, and 6). The simulated (using p*K*_a_ of compound and Curtipot software^41^) molar fraction of ionic forms at pH 5.2 is BPB^2−^ 0.979, BPB^−^ 0.021, BPB 0.00 (Fig. 4b).

**Fig. 7:**
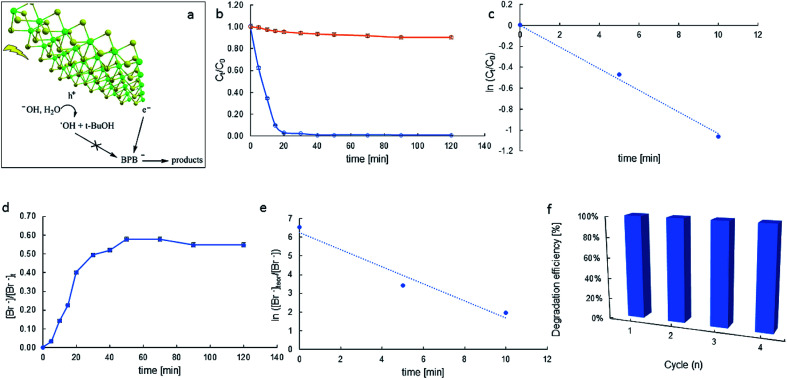
Schematic description of BPB photocatalytic degradation (a), BPB decay in time in the photocatalytic process (blue), and photolysis (orange) (b); *k*_app_ determination (c); bromide production in time (d); apparent rate constant (*k*_app_) for bromide generation determination (e); reusability of catalyst (f).

The progress of the photodegradation reaction was monitored by recording changes in the concentration of the BPB solution at regular intervals. Fig. 7 represents the results for the photocatalytic degradation of the BPB dye using MoS_2_ as the photocatalyst.

Almost over the entire time region, the substrate decays obey the pseudo-first-order kinetics eqn (11):11
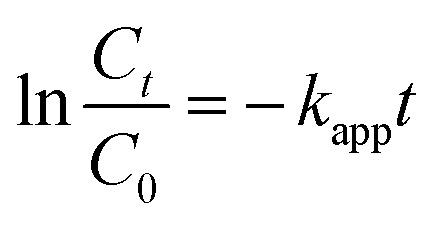
where *k*_app_ is the apparent rate constant; *C*_0_ and *C*_*t*_ are the initial concentration and concentration at time *t*.

The depletion of BPB concentration occurs mainly due to the photocatalytic properties of the synthesised materials and not because of the photolysis of the dye (Fig. 7b). For MoS_2_, the degradation efficiency of the BPB dye was very high and was found to be 100% for a time of 40 min.

A plot of ln(*C*/*C*_0_) *versus* time yields a straight line where *k*_app_ is the slope (see Fig. 7c). Photocatalytic activities of the catalysts were estimated by monitoring the decomposition of dye in the catalyst suspension upon illumination. The half-lives of decay (*t*_1/2_) were 6.7 min and the apparent rate constants (*k*_app_) calculated from the plots were 103.7 × 10^−3^ min^−1^.

The efficacy of catalysis depends on the catalyst–substrate interactions. The negative zeta potential of our molybdenite particles (*ca*. −14.4 mV) suggests anions in the Stern layer. Thus, in particular, in reaction conditions, the BPB-dianion (BPB^2−^) can be present in the vicinity of the MoS_2_ surface, probably near its Mo-built metallic edge. It allows the surface-substrate electron transfer, and reductive debromination of BPB^2−^ and relatively high *k*_app_, indicating the catalytic activity of MoS_2_.

Observed debromination can indicate the reducing pathway of BPB degradation. The release of bromide in time depicts Fig. 7d. Electron transfer-induced dehalogenation of halogenated organic compounds is a common mechanism in their redox chemistry.^42^ There are cases where electron transfer to a neutral precursor leaves the resulting radical ion in an electronic ground state that is dissociative. The process is called dissociative attachment.^43^ Following an electron transfer event, which is rapid on the timescale of nuclear motion, the ion relaxes along the dissociation coordinate, breaking one or more bonds. For aromatic halides, the reductive cleavage is believed to occur in a two-step mechanism (RX + e^−^ → RX^˙^ → R^˙^ + X^−^) with the transient formation of a radical anion (RX^˙−^). In particular, the accelerated degradation of BPB indicates the reductive debromination to be very efficient (*k*_app_ = 457.4 × 10^−3^ min^−1^, Fig. 7e).

The observed dye degradation occurs mainly as a result of reactions with the present on the catalyst surface e^−^ and h^+^, since hydroxyl radicals (˙OH) in the bulk of the solution are scavenged by *t*-BuOH, while saturation with argon eliminates oxygen in the solution and the potential formation of superoxide radicals (O^2˙−^). This confirms that electron mobility plays a major role in the photocatalytic degradation of BPB. It is assumed that the radicals formed as by-products of *t*-BuOH reaction with OH and H radicals are neutral.^44^

The MoS_2_ layer structure is favourable for the separation of charge carriers. The presence of a positive and negative charge density on the Mo and S terminated surfaces, respectively, produces a polarisation effect across these nanosheets, which causes an effective separation of the electron–hole pair and facilitates the movements of a photogenerated electron across the nanosheets. In photocatalytic oxidation processes, photogenerated holes in the valence band play a significant role. The valence band edge potential for MoS_2_ was estimated to be 1.6 V, which is not oxidative enough to generate hydroxyl radicals (^˙^OH). In addition, the reaction conditions were chosen so as (presence of *t*-BuOH, argon purging) to eliminate the oxidising agents such as hydroxyl radicals and/or superoxide radicals. The oxidation potential of BPB is +0.78 V,^45^ so BPB could be oxidised by holes. This process may be limited by the repulsion between the BPB and the catalyst surface.

However, recombination of the electron–hole pair is prevented by the presence of BPB molecules. These molecules can interact strongly with the generated electrons. Since the surface of the catalyst is negatively charged and the BPB molecule is negatively charged, strong repulsive forces occur there. Simultaneously, a reaction of BPB with a photocatalytically generated electron was observed. It suggests an interaction between the surface of the BPB molecule and the catalyst by relatively weak London forces.^46^

**Scheme 1:**
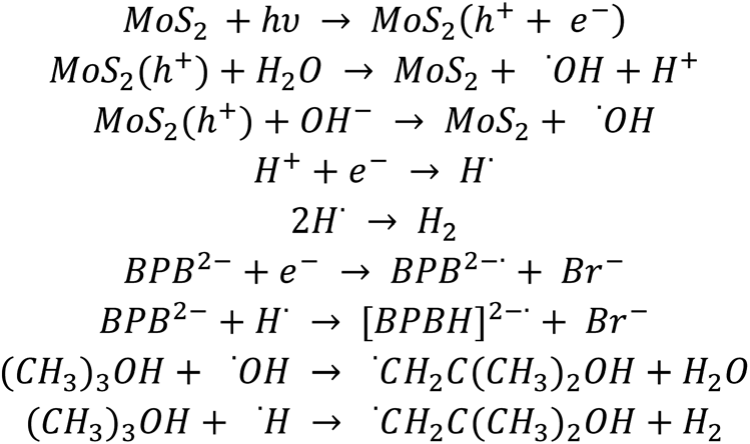
Proposed reaction path for the BPB degradation.

Based on the obtained kinetic results and simulation of the kinetics of the BPB degradation reaction, a suitable reaction path for the degradation of BPB has been proposed, as shown in Scheme 1:

Since the Kinetiscope™ software does not provide automatic fitting ability, we worked through trial and error. The best match of BPB decay, presented in Fig. S2,† was obtained immediately for the rate constants (*k*_(BPB+e)_ and *k*_(BPB+H)_) equal to 1.1 × 10^10^ and 2 × 10^9^ mol dm^−3^, respectively, while the product of luminous flux and quantum yield *i.e*. ‘hole and electron production yield’ was set equal to concentration of the catalyst in the suspension. The best match of Br^−^ formation yield was obtained when the rate of electron with secondary, partially dehalogenated products was set proportional to the number of remaining bromine atoms in the molecule (*i.e*. 3/4, 1/2 and 1/4 of *k*_(BPB+e)_).

The reusability of the catalysts is an important aspect for practical applications, and was evaluated for four successive runs. Four runs of BPB degradation reactions were investigated. As shown in Fig. 7f, the degradation rates after 60 min reached 99.3, 97.5, 93.1, and 89.9%, respectively. These results show layered MoS_2_ has high photocatalytic efficiency and good recycling performance.

Several articles have been written regarding the degradation of dyes. The BPB is a convenient model compound due to its high water solubility and the possibility of degradation in both oxidation and reduction processes. However, most of the works on BPB degradation describe oxidative processes.

Shah T. *et al.*^47^ in their study examined the photodegradation of BPB dye in an aqueous medium by graphene nanoplates-supported titanium dioxide (TiO_2_/GNP). The photodegradation study was performed as a function of time, and it was found that the photodegradation of dye in an aqueous medium increased as the time duration of UV irradiation increased. It was found that about 86% of dye was degraded within 8 h. The degradation of bromophenol blue is pH dependent and the rate of photodegradation increased with increasing solution pH, which could be due to the formation of more hydroxyl radicals at higher pH. The degradation of dye at pH 2, 4, 6, and 8 was about 70, 79, 90, and 95%, respectively. There were no data on the degradation reaction rate constants in the article.

Shah U. *et al.* in their recent study^48^ researched BPB photocatalytic degradation on two catalysts: zinc sulphide (ZnS) and tin-doped zinc sulphide (Sn–ZnS). About 86% and 96% dye degradation was observed in 300 min. The rate of dye degradation was found to increase with the increase in temperature (up to 70 °C) and pH (9.5). The recyclability study showed that both pure ZnS and Sn–ZnS NPs could be reused for the degradation of BPB. The mechanism proposed by Shah *et al.* describes the degradation of BPB under oxidative conditions. The photocatalytic degradation process was carried out in the temperature range from 40 to 70 °C. The obtained pseudo-first-order rate constants were in the range 4.7 × 10^−3^–11.4 × 10^−3^ min^−1^ and 5 × 10^−3^–14.5 × 10^−3^ for ZnS and Sn–ZnS, respectively.

Razavi *et al.*^49^ investigated the photocatalytic ability of Ti_*x*_Ni_*y*_La_*m*_O_*z*_ to degrade bromophenol blue in their recent work. The results show this catalyst can remove BPB from the aqueous solution. The authors tested several parameters influencing the rate of degradation. The resulting pseudo-first rate reaction constants were in the range of 0.7 × 10^−3^–7 × 10^−3^ min^−1^. The degradation process was carried out under oxidative conditions.

The photocatalytic activities of the fluorinated TiO_2_ composites with carbon nanotubes (CNTs–TiO_2_–F) were evaluated with BPB in Dlamini *et al.* article.^50^ Rapid photocatalytic degradation of BPB by CNTs–TiO_2_–F was observed with a percentage removal of 98% of BPB at 20 min of illumination. The as-prepared TiO_2_ showed the least photoactivity of all four catalysts, degrading up to 85% of BPB at 140 min. CNTs–TiO_2_ and TiO_2_–F, degraded BPB up to 94% in140 min irradiation time. The reaction kinetics show the reactions followed a pseudo-first order, increasing from 17.8 × 10^−3^ min^−1^ (TiO_2_) to 67.3 × 10^−3^ min^−1^ (CNTs–TiO_2_–F). The photodegradation mechanism was oxidative.

The degradation processes shown in papers^47–50^ rely on photo-oxidation undergoing upon the UV-irradiation of highly-engineered nanomaterials. While our approach, based on photo-reduction, offers similar effectiveness of defluorination on material absorbing both UV and visible light. We believe that our methodology of layered MoS_2_-synthesis sets up a space for further modification and improvement of in-use performance.

## 4 Conclusions

We have shown a feasible method for synthesising layered MoS_2_. The proposed method resulted in the improvement of the catalytic properties of MoS_2_ such as light harvesting, avoiding fast charge recombination, and creating numerous appropriate sites for electron transfer. The morphology, optical properties, surface properties and photocatalytic features of MoS_2_ were investigated.

The photocatalytic efficiency of the material was evaluated for the degradation of bromophenol blue dye. The degradations were conducted under reductive conditions (argon purging, hydroxyl radical scavenging). To propose the sequence of reactions taking place during the degradation process, simulations of the reaction kinetics were performed. Our work shows that MoS_2_ is a highly efficient photocatalyst for the reduction processes. After four cycles, the removal effectiveness was still as high as 89.9%

From the presented studies, it can be concluded that MoS_2_ may serve as an effective photocatalyst for environmental remediation. These studies can help in the development of cheap and effective water and wastewater treatment using advanced reduction processes (especially for the degradation of polyhalogenated arenes).

## Author contributions

Joanna Kisała: conception and design of study, analysis and interpretation of data, writing original draft. Ana M. Ferraria: data acquisition, analysis and interpretation of data. Nataliya Mitina: synthesis of material. Bogumił Cieniek: XRD data acquisition, analysis and interpretation of data. Piotr Krzemiński: SEM, AFM data acquisition, analysis and interpretation of data. Dariusz Pogocki: kinetic simulations data acquisition, analysis and interpretation of data, Roman Nebesnyi: analysis and interpretation of data, Oleksandr Zaichenko: analysis and interpretation of data, Yaroslav Bobitski: analysis and interpretation of data, group supervising.

## Conflicts of interest

There are no conflicts to declare.

## Supplementary Material

RA-012-D2RA03362C-s001
