# Supplementary material for: Photocatalytic activity of layered MoS2 in the reductive degradation of bromophenol blue
Source: RSC Adv. 2022 Aug 11;12(35):22465–75. doi: 10.1039/d2ra03362c (PMC9366594; doi:10.1039/d2ra03362c)
Supplement: RA-012-D2RA03362C-s001 [file RA-012-D2RA03362C-s001.pdf]

Electronic Supplementary Material (ESI) for RSC Advances.
This journal is © The Royal Society of Chemistry 2022

Photocatalytic activity of 2D-MoS_2 in the reductive degradation of bromophenol blue.

SUPPLEMENTARY

Table S1. Qualitative and quantitative analysis of 2D-MoS_2

	BE (eV)	At. Conc. (%)	Assignments
S $2p_{3/2}$<br>S $2p_{1/2}$	162.6<br>164.0	2.08<br>1.04	MoS_2
S $2p_{3/2}$<br>S $2p_{1/2}$	169.2<br>170.4	5.37<br>2.69	SO_4^{2-}
Mo $3d_{5/2}$<br>Mo $3d_{3/2}$	229.6<br>232.7	0.92<br>0.61	MoS_2
Mo $3d_{5/2}$<br>Mo $3d_{3/2}$	233.7<br>236.8	2.21<br>1.47	Mainly $Mo(SO_4)_3$; MoO_3 (residual); $Mo(CO_3)_3$ (residual)
C 1s	284.7	21.53	sp^2 (and sp^3 at 285 eV)
	286.2	10.06	C-N and C-O-C (or C-O)
	288.6	2.43	CO_3^{2-}
	291.1	1.57	π- π *
O 1s	528.8	0.71	MoO_3
	532.1	34.11	SO_4^{2-} (and O=C, residual)
	533.9	5.71	O-C
	537.5	1.69	H_2O
Na 1s	1071.8	2.65	Na^+
S 2s	226.7	—	MoS_2
	233.2	—	SO_4^{2-}
N 1s (overlapping Mo $3p_{5/2}$)	401.9	3.15	N from hydrazine chloride (no chlorine is detected)
Atomic ratios			*Observations*
$S_{(SO_4^{2-})}/S(S^{2-})$		2.58	S(sulfate)/S(sulfide) for S 2p
$S(S^{2-})/Mo_{(3d,\ 229.6;\ 232.8)}$		2.03	As expected
$[S_{(SO_4^{2-})} + C_{(CO_3^{2-})} + O_{(O^{2-})}]$ $/Mo_{(3d,\ 233.8;\ 236.8)}$		3.05	Compatible with $Mo(SO_4)_3$, $Mo(CO_3)_3$ and MoO_3
Oxidized species / O		1.02	
$O_{532.1}/S_{(SO_4^{2-})}$		4.08	As expected (subtracting O=C contribution)

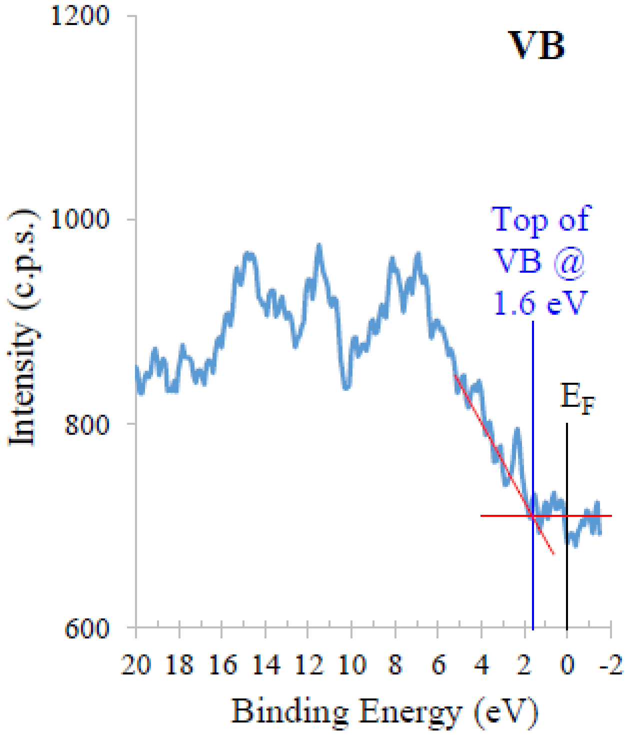

Figure S1. XPS Valence Band. The spectrum was smoothed (with a linear Savitzky-Golay function with Kernel width of 3), corrected from the charge shift, and the source satellites were subtracted.

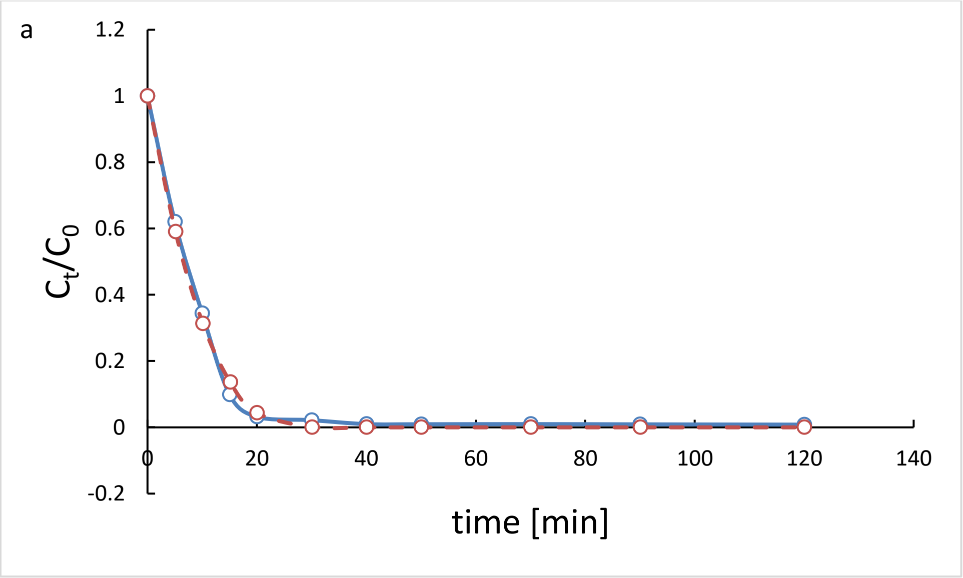

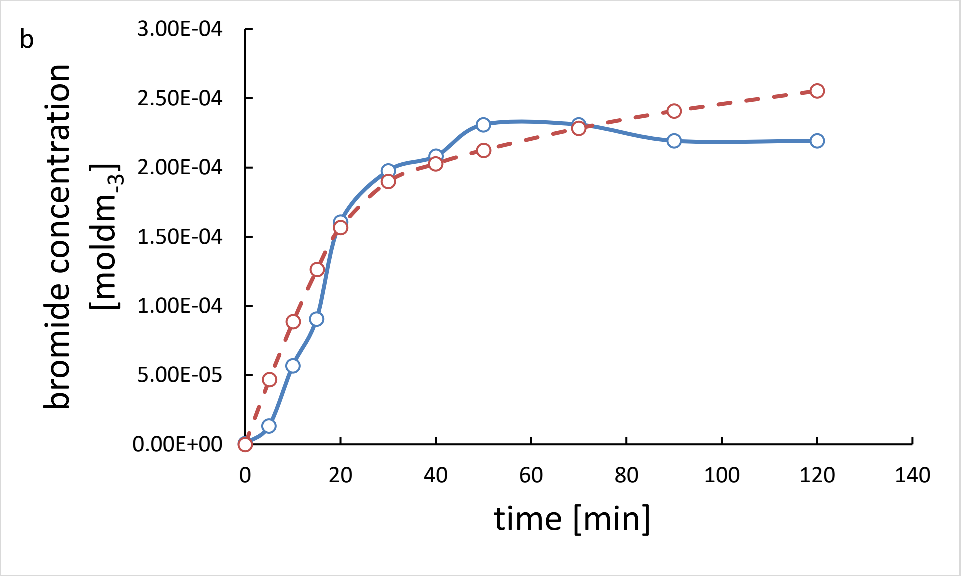

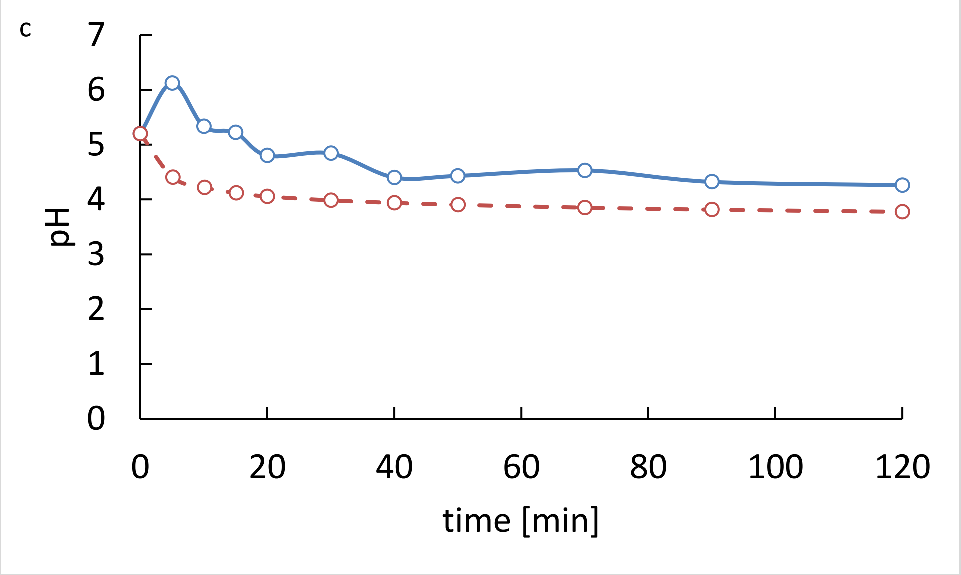

Figure S2. Comparison of the obtained and simulated results (actual results - blue, simulated results - red) of BPB decay (a), bromide production (b), pH changes of the reaction mixture (c) over time. The simulation was performed based on the proposed mechanism.

Table S2. The rate constants of reactions considered for the kinetic simulation of PFOA decomposition under ionizing radiation in N_2O-saturated aqueous solutions [1,2].

No.	Reaction	Rate constant [$dm^3\ mol^{-1}\ s^{-1}$]
1.	$2\ e_{aq}^{-} \rightarrow H_2 + 2\ OH^{-}$	1.1×10^{10}
2.	$e_{aq}^{-} + H^{\bullet} \rightarrow H_2 + OH^{-}$	2.5×10^{10}
3.	$e_{aq}^{-} + {}^{\bullet}OH \rightarrow OH^{-}$	3.0×10^{10}
4.	$e_{aq}^{-} + H^{+} \rightarrow H^{\bullet}$	2.3×10^{10}
5.	$2\ H^{\bullet} \rightarrow H_2$	1.55×10^{10}
6.	${}^{\bullet}OH$ + tert-Butanol $\rightarrow H_2O$	6×10^{8}
7.	$H^{\bullet}$ + tert-Butanol $\rightarrow H_2O$	1×10^{5}
8.	$H^{\bullet} + {}^{\bullet}OH \rightarrow H_2O$	7.0×10^{9}
9.	$H^{\bullet} + H_2O \rightarrow H_2 + {}^{\bullet}OH$	10
10.	$H^{\bullet} + OH^{-} \rightarrow e_{aq}^{-} + H_2O$	2.2×10^{7}
11.	e_{aq}^{-} + BPB $\rightarrow$ products + Br^{-}	1.1×10^{10}
12.	$H^{\bullet}$ + BPB $\rightarrow$ products + Br^{-}	2.9×10^{9}

References

[1] G.V. Buxton, C.L. Greenstock, W.P. Helman, A.B. Ross. 1988 *J. Phys. Chem. Ref. Data*, **17** 513-886.

[2] A.J. Elliot, A. 1989 *Radiat. Phys. Chem.* **34** 753-758.
